# *Hiptage yangshuoensis* (Malpighiaceae), a new species on karst hills close to Lijiang River, Guangxi, China, based on molecular and morphological data

**DOI:** 10.1002/ece3.70099

**Published:** 2024-07-31

**Authors:** Ren‐Fen Wang, Yao Ning, Xiao‐Juan Li, Ke Tan, Khang Sinh Nguyen

**Affiliations:** ^1^ Guangxi Key Laboratory of Plant Conservation and Restoration Ecology in Karst Terrain Guangxi Institute of Botany, Guangxi Zhuang Autonomous Region and Chinese Academy of Sciences Guilin Guangxi China; ^2^ Institute of Ecology and Biological Resources Vietnam Academy of Science and Technology Ha Noi Vietnam; ^3^ Graduate University of Science and Technology, Vietnam Academy of Science and Technology Hanoi Vietnam

**Keywords:** flora of Guangxi, karst landform, taxonomy, the Lijiang River

## Abstract

*Hiptage yangshuoensis* K.Tan & K.S.Nguyen, a new species of *Hiptage* collected from a karst cliff close to the Lijiang River, Northeast of Guangxi Zhuang Autonomous Region, China, is described and illustrated based on molecular and morphological data. *Hiptage yangshuoensis* shares some morphological similarities with the *H. multiflora* F.N.Wei, but easily distinguished by its long pedicels with articulate at top, one large calyx gland, oblanceolate middle wing and lanceolate lateral wings of samara, and young branch covered rusty sericeous. The new species status is also supported by molecular phylogenetic analyses based on nuclear ribosome internal transcribed spacer (nrITS), which showed distinct systematic distinctiveness from the most morphologically similar species, *H. multiflora*.

## Graphical abstract

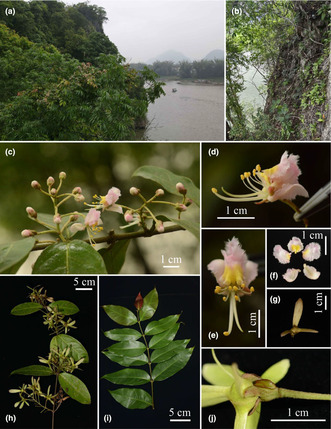

## 1 INTRODUCTION

*Hiptage* Gaertn. (Gaertner, 1791) is the largest genus of Malpighiaceae in Old World, with 47 accepted species (POWO, 2024; Zhang et al., 2023). These woody shrubs or lianas are native to tropical Asia's shrub forests or valleys on limestone hills or riverbanks, notably in regions like the Indo‐China Peninsula, Malay Archipelago, and Southern China (Chen & Funston, 2008; Dong et al., 2020; Hô, 1992; Ren, 2015; Sirirugsa, 1991; Srivastava, 1992; Tan et al., 2019; Wei, 2018, 2023; Wei et al., 2022; Yang et al., 2018; Zhang et al., 2023). Leaves opposite, leathery or subleathery, entire, usually with two basal glands on abaxial surface; stipules minute, glandlike, or absent. Flowers white, sometimes pinkish, fragrant, bisexual, zygomorphic. The style is deflected away from the floral axis, either to the left in left‐styled flowers or to the right in right‐styled flowers, and characterizes these as “mirror‐image flowers” (Ren et al., 2013). Stamens are 10 in number and unequal; one, much larger than the others, acts as the “pollinating anther” and serves reproductive functions, while another serves as the “feeding anther,” providing rewards to pollinators (Ren et al., 2013). Samaras usually have  3 wings; the abaxial wing is long and erect, whereas the other 2 lateral wings are short and spread outward (Chen & Funston, 2008; Sirirugsa, 1991). In recent years, five new species of *Hiptage* have been described in Southwestern China (Dong et al., 2020; Tan et al., 2019; Yang et al., 2018; Zhang et al., 2023).

Current molecular evidence suggests that the *Hiptage* is monophyletic, with *Chlorohiptage* T.V.Do, T.A.Le & R.F.Almeida from Vietnam identified as its sister group (Do et al., 2024). Although there is only a single marker (ITS) phylogenetic study of *Hiptage* and many clades with weak support, it is clear that *H. stellulifera* Arènes is the most basal species within the genus (Almeida & van den Berg, 2022; Tan et al., 2019).

During our biodiversity surveying along the Lijiang River, Guilin City, Northeast of Guangxi Zhuang Autonomous Region, China, we collected specimens from a distinct morphotype of *Hiptage* growing on a karst cliff close to the Lijiang River. After detailed analyses of morphological characters and molecular data, along with comparisons of specimens, we found that the species of our collection is most morphologically similar to *H. multiflora* F.N.Wei (Chen & Funston, 2008). However, it differs in the lanceolate lateral wings of samara, young branch covered rusty sericeous, and pedicel articulate at top, both of which are critical morphological traits for species taxonomy in *Hiptage* (Chen & Funston, 2008; Ren, 2015). Therefore, we describe it as a new species, depicted and illustrated here.

## 2 MATERIALS AND METHODS

### 2.1 Taxonomy

**FIGURE 1:**
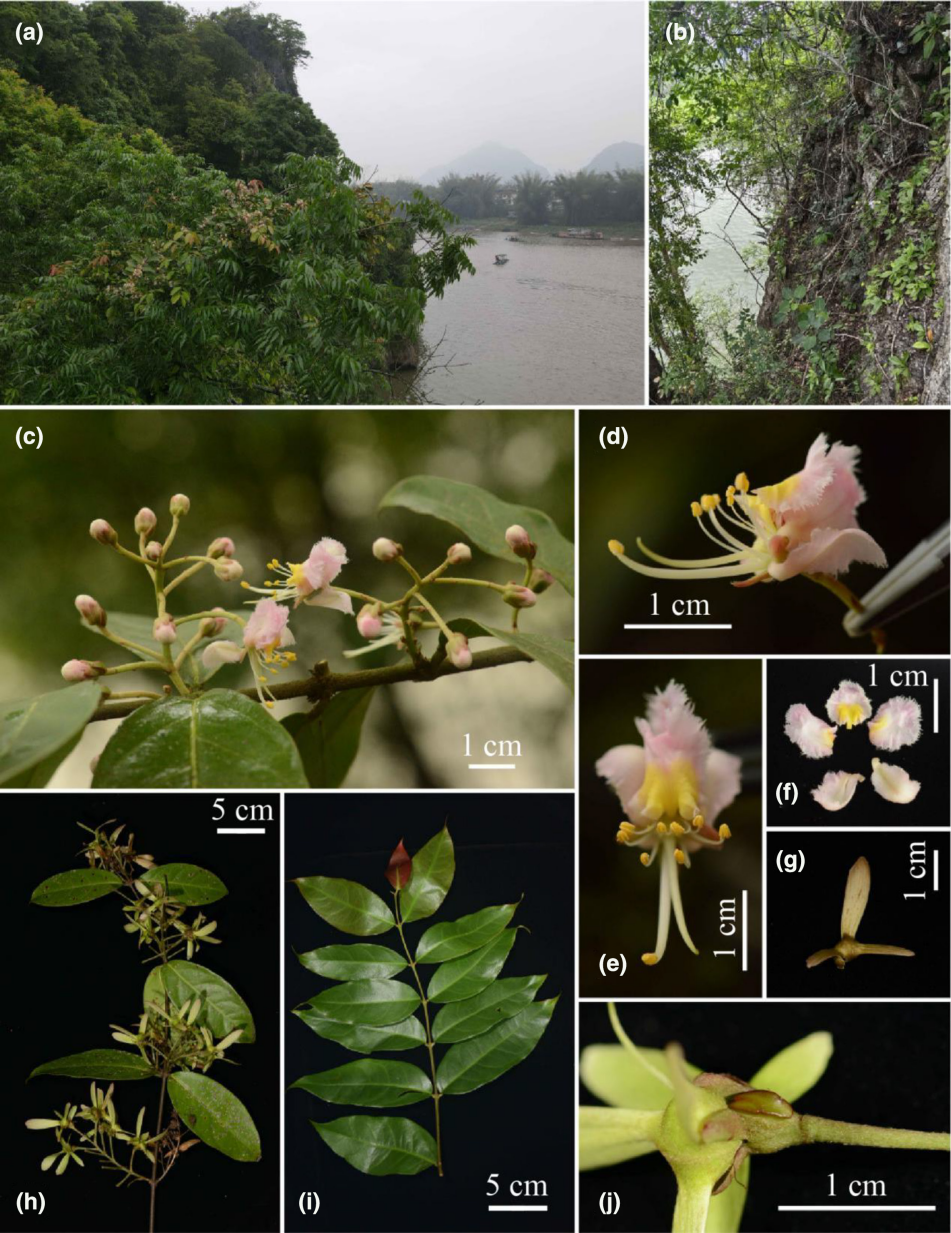
*Hiptage yangshuoensis* (a) habitat, (b) habit, (c) inflorescence, (d) flower in side view, (e) flower in frontal view, (f) petals, (g) samara, (h) fruiting branch, (i) branchlets, (j) calyx gland (showing articulate at top of pedicel).

Morphological analysis of the new *Hiptage* species was conducted using fresh or preserved specimens, with measurements of specific traits averaged across five individuals. In‐field photographs captured the plants’ and its flowers' characteristics (Figure 1). Comparative morphological evaluations were conducted with type specimens of *Hiptage* from herbaria (IBK, KUN, IBSC, and HUTB), utilizing protologues and herbarium samples alongside digital resources from JSTOR Global Plants (http://plants.jstor.org) and the Chinese Virtual Herbarium (http://www.cvh.ac.cn), as detailed in Table S1, and relevant taxonomic literature (e.g., Tan et al., 2019; Yang et al., 2018; Zhang et al., 2023). The morphological terminology follows Niedenzu (1924), Jacobs (1955), Anderson et al. (2006), Chen and Funston (2008), and Almeida and Morais (2022). The conservation status assessments of the new species *H. yangshuoensis* were based on the International Union for Conservation of Nature guidelines (IUCN Standards and Petitions Committee, 2024).

### 2.2 Phylogenetic analysis

To verify the taxonomic status of the new species within *Hiptage*, we conducted a phylogenetic analysis using the nuclear ribosomal internal transcribed spacer (ITS) region. We downloaded ITS sequence data for 39 samples from GenBank (Table S2) encompassing 21 species of *Hiptage*, based on the study of Almeida and van den Berg (2022), four species from the Tetrapteroid clade serving as outgroups [i.e., *Callaeum psilophyllum* (A.Juss.) D.M.Johnson, *Heteropterys brunnea* R.Sebast. & Mamede, *Niedenzuella multiglandulosa* (A.Juss.) W.R.Anderson, and *N. stannea* (Griseb.) W.R.Anderson] (Table S2). Dried leaf material of the proposed new species was collected from the type locality in Fuli Town, Yangshuo County (Guangxi, China). Two samples from the new species and one sample from *H. multiflora* were newly sequenced in this study to provide molecular evidence (Table S2). Total genomic DNA was isolated from dried leaf samples using a CTAB protocol adapted from Doyle and Doyle (1987). PCR amplification, in 25‐μL reactions, comprised 1 μL of sample DNA, 12.5 μL of 2 × Taq PCR Master Mix (Aidlab Biotechnologies Co., Ltd), and 1 μL of each primer (10 μmol/mL), with double distilled water making up the remainder. Amplification of the ITS region utilized primers ITS17SE and ITS26SE as per Sun et al. (1994). The amplification regime involved a 5‐min initial denaturation at 94°C, then 35 cycles of 40 s at 94°C, 20 s at 69°C, and 1 min at 72°C, and concluded with a 10‐min extension at 72°C. The resulting PCR products were bidirectionally sequenced on an ABI3730XL platform.

ITS fragment sequencing involved bidirectional evaluation using PhyDE for accuracy (Müller et al., 2010), followed by manual alignment in MEGA v.7 (Kumar et al., 2016). Using ModelFinder (Kalyaanamoorthy et al., 2017), the SYM + G4 model and the SYM + R2 model were selected as the optimal substitution models for maximum likelihood (ML) analysis and Bayesian inference (BI), respectively. Bayesian inference utilized MCMC simulations for 10 million generations in MrBayes v3.2.5 (Ronquist et al., 2012), discarding the initial 25% of trees as burn‐in, while ML analysis was executed in IQ‐TREE v.2.0.6 (Nguyen et al., 2015) with 1000 bootstrap replicates. Both MrBayes and IQ‐TREE analyses were integrated into PhyloSuite 1.2.2 (Zhang et al., 2020). The resultant tree was displayed using FigTree v.1.4.3 (http://tree.bio.ed.ac.uk/software/figtree/).

## 3 RESULTS AND DISCUSSION

**FIGURE 2:**
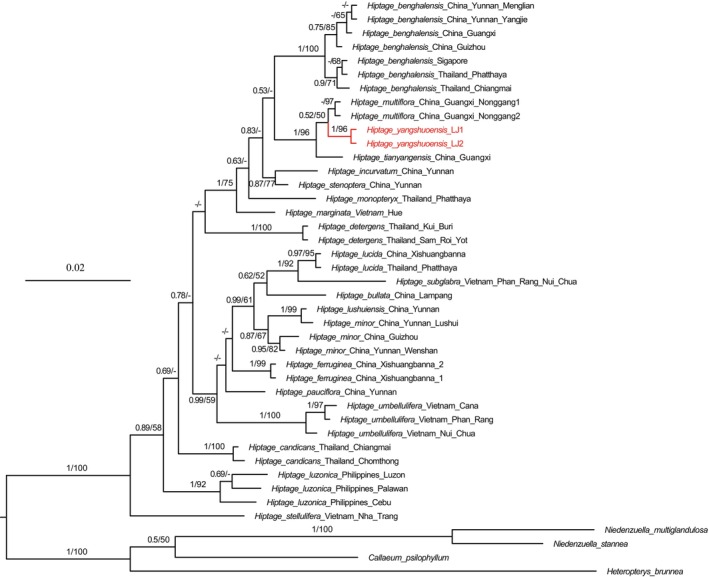
Molecular phylogenetic tree of *Hiptage* based on ITS sequences. Bayesian posterior probability (PP) and ML bootstrap values (BS) are shown above branches as PP/BS (only shown if PP > 0.5 and BS > 50).

**FIGURE 3:**
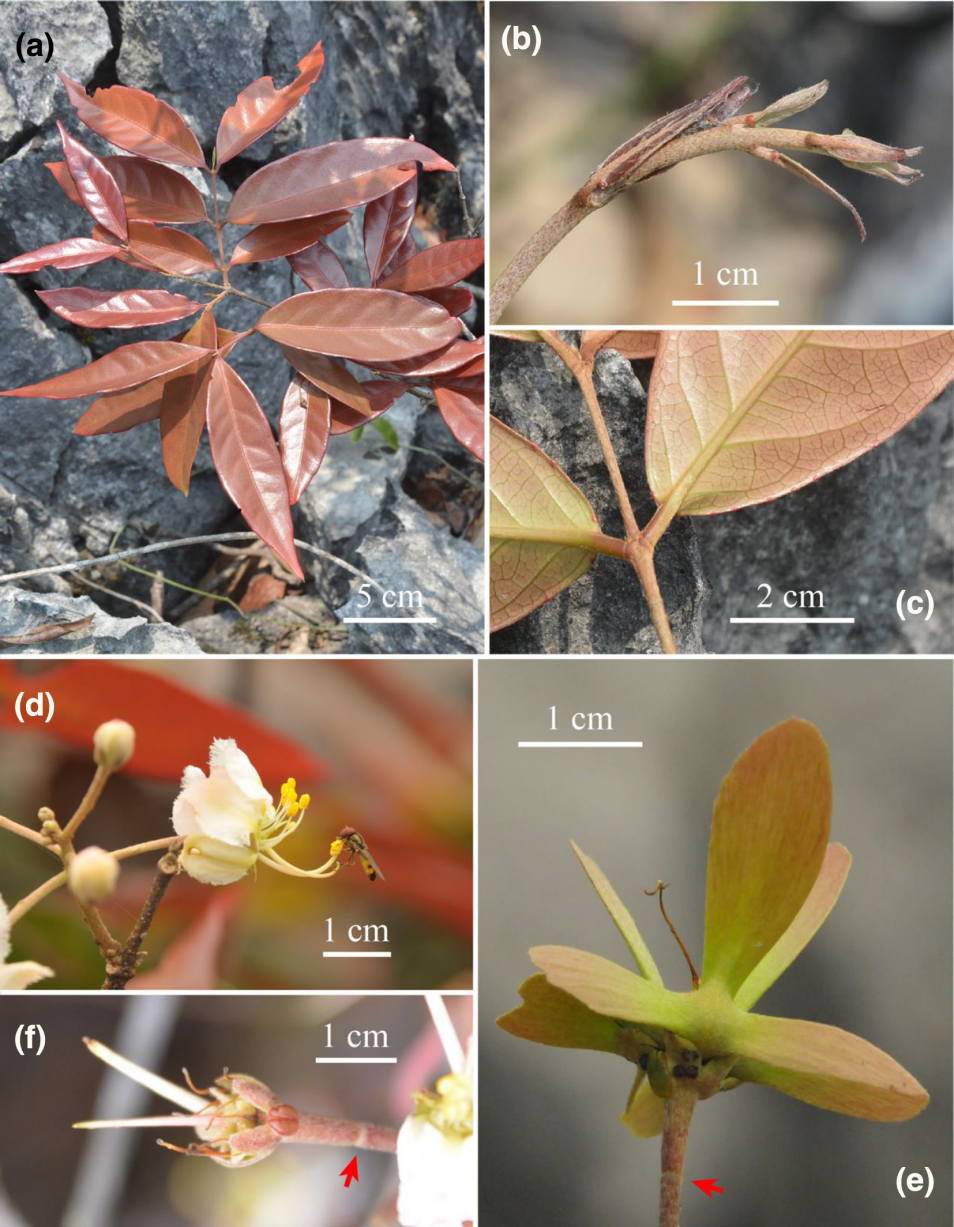
*Hiptage multiflora* (a) branchlets, (b) young branch covered white to rusty sericeous, (c) leaf glands, (d) flower in side view, (e) samara, (f) calyx glands initially 2, merge at the base into one as they mature. The arrow indicates the articulate position on the pedicel.

The aligned matrix of ITS sequences consisted of 687 bp, of which 466 sites were identical, 123 (17.9%) were parsimony informative, and 98 parsimony‐uninformative variable characters. The phylogenetic analysis showed that *Hiptage* is a monophyletic group (PP/BS = 1/100), with *H. stellulifera* (PP/BS = 1/100) being the first lineage to diverge, consistent with previous studies (Almeida & van den Berg, 2022; Tan et al., 2019; Zhang et al., 2023) (Figure 2). The two samples of the proposed new species, *H. yangshuoensis*, formed a clade with strong support (PP/BS = 1/96), which is sister to a subclade with weak support, including *H. multiflora* (PP/BS = 0.52/50) (Figure 2). The results of molecular phylogenetics align with morphological observations, with the new species closely resembling *H. multiflora* due to the pink flower, 1 sepal gland, and sepal gland not decurrent to pedicel. However, *H. yangshuoensis* is distinguished by its samara middle wing oblanceolate and lateral wing lanceolate (vs. obovate and oblong), young branch covered rusty sericeous (vs. white to rusty puberulent), and pedicel articulate at top (vs. at middle). Other features, like pedicels, differentiate the two species, as detailed in Table 1 and Figure 3.

**TABLE 1:** Morphological comparison of key characteristics in *H. yangshuoensis* and *H. multiflora.*

Character	*H. yangshuoensis*	*H. multiflora*
Leaf blade	Ovate, 2 glands	Oblong, more than 2 glands
Young branch	Rusty sericeous	White to rusty puberulent
Pedicel	1.5–2.3 cm long, articulate at top, green, rusty sericeous	ca. 1 cm long, articulate at middle, red, white puberulent
Calyx glands	1	Initially 2, merge at the base into one as they mature
Middle wing of Samara	Oblanceolate, 0.6–0.7 cm wide	Obovate, 1.0–1.4 cm wide
Lateral wings of Samara	Lanceolate	Oblong

**FIGURE 4:**
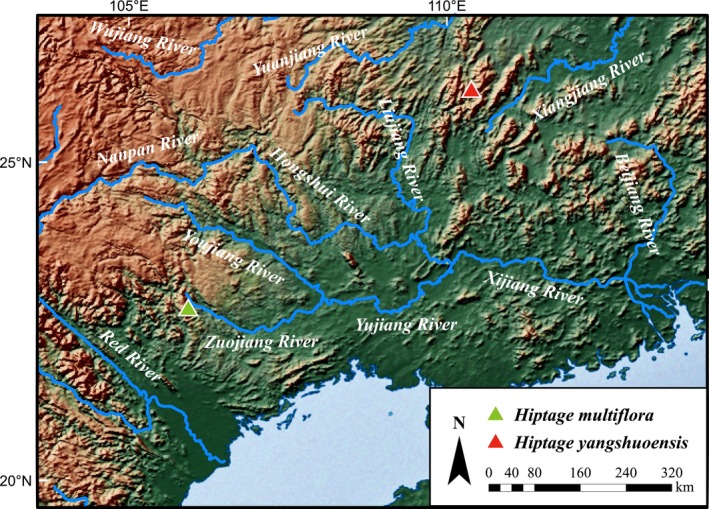
Distribution map showcasing the new species, *Hiptage yangshuoensis*, alongside its similar species, *H. multiflora*. [Correction added September 03 2024, after first online publication: Figure 4 has been updated.]

*Hiptage yangshuoensis* is morphologically similar to *H. multiflora*. However, the new species differs by several key morphological characters (summarized in Table 1) and molecular data (Figure 2). Moreover, the *H. yangshuoensis* is found in northeastern Guangxi (Figure 4), whereas *H. multiflora* inhabits the southwestern region of Guangxi, with over 500 kilometers separating their habitats. This significant distance, along with the distinctive “terrestrial islands” ecosystem created by the karst landscape, readily elucidates the speciation phenomenon observed within these karst species (e.g., Ke et al., 2022; Wang et al., 2017). Presently, this new species marks another recent discovery at the northern boundary of the genus's distribution range, succeeding the identification of several *Hiptage* species in northwestern Yunnan (Dong et al., 2020; Tan et al., 2019; Zhang et al., 2023).

## 4 TAXONOMIC TREATMENT

*Hiptage yangshuoensis* K.Tan & K.S.Nguyen, sp. nov.

urn:lsid:ipni.org:names:**********

Figure 1

*Diagnosis*. *Hiptage yangshuoensis* differs from the closely related *H. multiflora* by the rusty sericeous (vs. white to rusty puberulent) young branch, pedicels 1.5–2.3 cm and articulate at top (vs. ca. 1 cm, at middle), 1 calyx gland (vs. initially 2, the calyx glands merge at the base into one as they mature), and samara with oblanceolate (vs. obovate) middle wing and lanceolate (vs. oblong) lateral wing.

*Type*. CHINA. Guangxi Zhuang Autonomous Region: Guilin City, Yangshuo County, Fuli Town, close to Lijiang River, 24°46′41″ N, 110°33′11″ E, 120 m a.s.l., 11 Apr. 2023, K. Tan23tk042201 (Holotype: IBK!; Isotype: IBK!; Paratype: IBK! IBK00414206).

*Description*. Woody shrubs; young branches rusty sericeous, hairs adpressed, older twigs glabrous, with white or greenish lenticels, rounded, coarse warts dotted. Leaves opposite; petiole ca. 0.5 cm long with yellowish brown eglandular sericeous; leaf blades ovate, 7.0–16.0 × 2.5–6.0 cm, coriaceous; densely rusty sericeous on abaxial surfaces when young, and glabrous at mature, base obtuse or broadly cuneate, margin entire or slightly undulate, apex acute or acuminate, abaxially often with 2 marginal glands near the base; lateral veins 5–8 pairs, prominent on both surfaces. *Thyrses*, terminal or axillary; main axis 3.5–6.0 cm long, rusty sericeous; peduncle 1.0–2.5 cm long, rusty sericeous, pedicel articulate at top; bracteoles ca. 0.5 cm long, lanceolate. *Flowers* pink; pedicels 1.5–2.3 cm long, articulate at top, green, rusty sericeous; sepals 5, 4.0–5.0 × 1.5–2.5 mm, ovate, apex obtuse, margin slightly involute, abaxially rusty sericeous, adaxially glabrous. *Calyx glands* 1, prominent, not decurrent to the pedicel, 3.8–4.0 × 1.2–1.4 mm, connate at the base. *Petals* 5, 0.7–1.2 × 0.6–1.0 cm, pink, basally yellow, extremely reflexed, suborbicular, margin ciliate, claws ca. 1 mm long, base subcordate to rounded, apex roundish, abaxially densely white sericeous, adaxially glabrous. *Stamens* 10, basally fused or free, glabrous, differing in size; one larger, filament 14–16 mm long, white, circinate, anther oblong, 1.2–1.5 × 0.7–1.0 mm; 9 smaller stamens, filament 4–6 mm long; anthers oblong, 1–1.1 × 0.4–0.6 mm. *Ovary* ca. 2 mm in diam., ovoid, rusty sericeous; style 1, white, 15–17 mm long, slightly curved upward, deflected either to the left or right side, glabrous; stigma apical. *Mericarps* 3, wings pink with green base, rusty sericeous, middle wing 2.3–2.7 × 0.6–0.7 cm, oblanceolate, apex roundish or lobed slightly; lateral wings 1.4–1.6 × 0.3–0.4 cm, lanceolate; areole 5–6 mm, approximately triangular. *Seeds* angular‐globose, 3–4 mm long, dark yellow or brown.

*Phenology*. Flowering in April, and fruiting from April to May.

*Etymology*. *Hiptage yangshuoensis* was discovered only near the Lijiang River in Yangshuo County, Guilin City, in the Guangxi Zhuang Autonomous Region of China. The specific epithet is derived from the name of the Lijiang River.

*Vernacular name*. Chinese: 阳朔风筝果 (yáng shuò fēng zhēng guǒ).

*Habitat and distribution*. *Hiptage yangshuoensis* is only known from the karst hill close to Lijiang River, at an elevation ca. 120 m, near Yangshuo County, northeast of Guangxi Zhuang Autonomous Region, China (Figure 4).

*Conservation status*. Since the only known population of *Hiptage yangshuoensis* is growing on a karst cliff close to the Lijiang River in the northeast Guangxi Zhuang Autonomous Region, we have not discovered the wild population outside of the abovementioned place, information known about the population status and natural distribution range of the new species is very limited. Currently, only about 10 individuals have been identified on the karst cliff by unmanned aerial vehicle (UAV). Therefore, we suggest that the new species *H. yangshuoensis* should be considered “Critically Endangered” [CR, B2a,b (iii, iv, v)] according to current IUCN Red List Categories and Criteria (IUCN Standards and Petitions Committee, 2024).

## AUTHOR CONTRIBUTIONS

**Ren‐Fen Wang:** Methodology (equal); software (equal); writing – original draft (supporting). **Yao Ning:** Data curation (supporting); formal analysis (equal); software (equal). **Xiao‐Juan Li:** Formal analysis (equal); methodology (equal). **Ke Tan:** Conceptualization (lead); resources (lead); writing – original draft (lead); writing – review and editing (equal). **Khang Sinh Nguyen:** Data curation (lead); writing – review and editing (equal).

## FUNDING INFORMATION

The Hainan Provincial Natural Science Foundation of China (422RC594), the Fund of Guangxi Key Laboratory of Plant Conservation and Restoration Ecology in Karst Terrain (No.22–035‐26), and the Project for Fundamental Research of Guangxi Institute of Botany (GZY24002 and GZY24006).

## CONFLICT OF INTEREST STATEMENT

The authors declare that there is no conflict of interest.

## Supporting information


Table S1.

## Data Availability

The sequences used in this study have been deposited in The National Center for Biotechnology Information (NCBI) database. GenBank accession numbers are provided in Table S2.

## References

[ece370099-bib-0001] Almeida, R. F. , & Morais, I. L. (2022). Morphology of Malpighiaceae from Brazil ‐ part 1 ‐ vegetative (pp. 1–44). Universidade Estadual de Goiás.

[ece370099-bib-0002] Almeida, R. F. , & van den Berg, C. (2022). Biogeography and character‐mapping of *Hiptage* (Malpighiaceae) corroborate Indochina's rainforests as one of the main sources of plant diversity in southeastern Asia. Nordic Journal of Botany, 4, e03464.

[ece370099-bib-0003] Anderson, W. R. , Anderson, C. , & Davis, C. C. (2006). Malpighiaceae. University of Michigan. http://herbarium.lsa.umich.edu/malpigh/index.html

[ece370099-bib-0004] Chen, S. K. , & Funston, A. M. (2008). Malpighiaceae. In Z. Y. Wu , P. H. Raven , & D. Y. Hong (Eds.), Flora of China (Vol. 11, pp. 132–138). Science Press, Beijing and Missouri Botanical Garden Press.

[ece370099-bib-0006] Do, V. T. , Lu, N. T. , Le, A. T. , Lam, M. X. T. , Trinh, X. T. , Deguine, J. P. , Hoang, T. T. , & de Almeida, R. F. (2024). *Chlorohiptage* (Tetrapteroids, Malpighiaceae), a distinct new genus endemic to Vietnam based on morphological and molecular data. Plant Ecology and Evolution, 157(2), 125–136.

[ece370099-bib-0007] Dong, S. , Tan, K. , & Ren, M. X. (2020). A new species of *Hiptage* (Malpighiaceae) from Nujiang gorge, southwest China. Nordic Journal of Botany, 38(3), e02436.

[ece370099-bib-0008] Doyle, J. J. , & Doyle, J. L. (1987). A rapid DNA isolation procedure for small quantities of fresh leaf tissue. Phytochemical Bulletin, 19, 11–15.

[ece370099-bib-0009] Gaertner, J. (1791). De Fructibus et Seminibus Plantarum (Vol. 2). Typis Guilielmi Henrici Schrammii, Tubinge. 169, tab. 116, f. 4.

[ece370099-bib-0010] Hô, P. H. (1992). Malpighiaceae. Câycó Viêtnam, 2(1), 424–434. [Illustrated flora of Vietnam].

[ece370099-bib-0011] IUCN Standards and Petitions Committee . (2024). Guidelines for using the IUCN red list categories and criteria. Version 16. Prepared by the Standards and Petitions Committee. https://www.iucnredlist.org/documents/RedListGuidelines.pdf

[ece370099-bib-0012] Jacobs, M. (1955). In C. G. G. J. Steenis (Ed.), Flora Malesiana Ser. 1, 5(2) Malpighiaceae (pp. 125–145). Noordhoff‐Kolff.

[ece370099-bib-0013] Kalyaanamoorthy, S. , Minh, B. Q. , Wong, T. K. F. , Haeseler, A. N. , & Jermiin, L. S. (2017). ModelFinder: Fast model selection for accurate phylogenetic estimates. Nature Methods, 14(6), 587–589. 28481363 10.1038/nmeth.4285 PMC5453245

[ece370099-bib-0014] Ke, F. , Vasseur, L. , Yi, H. , Yang, L. , Wei, X. , Wang, B. , & Kang, M. (2022). Gene flow, linked selection, and divergent sorting of ancient polymorphism shape genomic divergence landscape in a group of edaphic specialists. Molecular Ecology, 31(1), 104–118. 34664755 10.1111/mec.16226

[ece370099-bib-0015] Kumar, S. , Stecher, G. , & Tamura, K. (2016). MEGA7: Molecular evolutionary genetics analysis version 7.0 for bigger datasets. Molecular Biology and Evolution, 33(7), 1870–1874. 27004904 10.1093/molbev/msw054 PMC8210823

[ece370099-bib-0016] Müller, K. , Müller, J. , & Quandt, D. (2010). PhyDE: Phylogenetic Data Editor, version 0.9971. http://www.phyde.de/index.html

[ece370099-bib-0017] Nguyen, L. T. , Schmidt, H. A. , Von Haeseler, A. , & Minh, B. Q. (2015). IQ‐TREE: A fast and effective stochastic algorithm for estimating maximum‐likelihood phylogenies. Molecular Biology and Evolution, 32(1), 268–274. 25371430 10.1093/molbev/msu300 PMC4271533

[ece370099-bib-0018] Niedenzu, F. (1924). Malpighiaceae palaeotropicae II. Verzeichnis der Vorlesungen an der Akademie Zu Braunsberg Im Sommer. 1–19.

[ece370099-bib-0019] POWO . (2024). Plants of the world online. Facilitated by the Royal Botanic Gardens, Kew. Published on the Internet. http://www.plantsoftheworldonline.org/

[ece370099-bib-0020] Ren, M. X. (2015). The upper reaches of the largest river in southern China as an “evolutionary front” of tropical plants: Evidences from Asia‐endemic genus *Hiptage* (Malpighiaceae). Collectanea Botanica, 34, e003.

[ece370099-bib-0117] Ren, M. X. , Zhong, Y. F. , & Song, X. Q. (2013). Mirror‐image flowers without buzz pollination in the Asia‐endemic *Hiptage benghalensis* (Malpighiaceae). Botanical Journal of the Linnean Society, 173(4), 764–774.

[ece370099-bib-0021] Ronquist, F. , Teslenko, M. , van der Mark, P. , Ayres, D. L. , Darling, A. , Höhna, S. , Larget, B. , Liu, L. , Suchard, M. A. , & Huelsenbeck, J. P. (2012). MrBayes 3.2: Efficient Bayesian phylogenetic inference and model choice across a large model space. Systematic Biology, 61(3), 539–542. 22357727 10.1093/sysbio/sys029 PMC3329765

[ece370099-bib-0022] Sirirugsa, P. (1991). Malpighiaceae. In T. Smitinand & K. Larsen (Eds.), Flora of Thailand (Vol. 5, Part 3, pp. 272–299). The Forest Herbarium, Royal Forest Department.

[ece370099-bib-0023] Srivastava, R. C. (1992). Taxonomic revision of the genus *Hiptage* Gaertner (Malpighiaceae) in India. Candollea, 47, 601–612.

[ece370099-bib-0024] Sun, Y. , Skinner, D. Z. , Liang, G. H. , & Hulbert, S. H. (1994). Phylogenetic analysis of sorghum and related taxa using internal transcribed spacers of nuclear ribosomal DNA. Theoretical and Applied Genetics, 89(1), 26–32. 24177765 10.1007/BF00226978

[ece370099-bib-0025] Tan, K. , Zeng, H. L. , Dong, S. P. , & Ren, M. X. (2019). Molecular phylogeny of *Hiptage* (Malpighiaceae) reveals a new species from southwest China. PhytoKeys, 135, 91–104. 31849561 10.3897/phytokeys.135.37011 PMC6908513

[ece370099-bib-0026] Wang, J. , Ai, B. , Kong, H. , & Kang, M. (2017). Speciation history of a species complex of *Primulina eburnea* (Gesneriaceae) from limestone karsts of southern China, a biodiversity hot spot. Evolutionary Applications, 10(9), 919–934. 29151883 10.1111/eva.12495 PMC5680421

[ece370099-bib-0027] Wei, Y. G. (2018). The distribution and conservation status of native plants in Guangxi, China (p. 221). China Forestry Publishing House.

[ece370099-bib-0028] Wei, Y. G. (2023). Catalogue and red list of plant species in Guangxi (p. 483). China Forestry Publishing House.

[ece370099-bib-0029] Wei, Y. G. , Do, T. V. , & Wen, F. (2022). A checklist to the plants of northern Vietnam (pp. 251–252). China Forestry Publishing House.

[ece370099-bib-0030] Yang, B. , Ding, H. B. , Li, J. W. , & Tan, Y. H. (2018). Two new species of *Hiptage* (Malpighiaceae) from Yunnan, southwest of China. PhytoKeys, 110, 81–89. 10.3897/phytokeys.110.28673 PMC6232241 30429660

[ece370099-bib-0031] Zhang, D. , Gao, F. , Jakovlić, I. , Zou, H. , Zhang, J. , Li, W. X. , & Wang, G. T. (2020). PhyloSuite: An integrated and scalable desktop platform for streamlined molecular sequence data management and evolutionary phylogenetics studies. Molecular Ecology Resources, 20(1), 348–355. 31599058 10.1111/1755-0998.13096

[ece370099-bib-0032] Zhang, T. T. , Yang, S. Y. , Tan, K. , & Ren, M. X. (2023). A new species of *Hiptage* (Malpighiaceae) from northwest Yunnan (China) based on molecular and morphological data. PhytoKeys, 232, 45–57. 37705964 10.3897/phytokeys.232.106675 PMC10495827

