# Supplementary material for: Hiptage yangshuoensis (Malpighiaceae), a new species on karst hills close to Lijiang River, Guangxi, China, based on molecular and morphological data
Source: Ecol Evol. 2024 Jul 31;14(8):e70099. doi: 10.1002/ece3.70099 (PMC11290907; doi:10.1002/ece3.70099)
Supplement: Supplementary file 1 — Table S1. [file ECE3-14-e70099-s001.docx]

**Table S1** The list of type specimens has been checked in this study for *Hiptage* in China.

| Species Name | Type | Specimen Number | Collection Number | Collection Location | Collection Date | Herbarium |
| --- | --- | --- | --- | --- | --- | --- |
| *Hiptage acuminata* Wallich ex A. Jussieu | Holotype | K000739262 | - | India | - | K |
|  | Isosyntype | M0242669 | - | Pundua, India |  | M |
|  | Isosyntype | M0242670 | - | Pundua, India |  | M |
| *H. fraxinifolia* F. N. Wei | Type | IBK00190750 | 50482 | Hengxian, Guangxi | 8 May, 1957 | IBK |
| *H. benghalensis* | Type | BM000946556 | - | - | - | BM |
|  | Type | BM000946557 | - | - | - | BM |
|  | Isosyntype | MPU020097 | 4330 | Vietnam | - | MPU |
| *H. candicans* | Type | BM000796253 |  | Thailand | 9 March, 1910 | BM |
|  | Type | K000739304 |  | Thailand | 9 March, 1910 | K |
|  | Type | C10014223 |  | Samrong Tong, Cambodia | 20 December, 1985 | C |
|  | Type | BM000796254 |  | Thailand | 9 March, 1910 | BM |
| *H. incurvatum* K.Tan & M.X.Ren | Type | - | 2019033107 | Yangbi, Yunnan | 31 March, 2019 | HUTB |
|  | Isotype | 1347924 | 2019033108 | Yangbi, Yunnan | 31 March, 2019 | KUN |
| *H. lanceolata* Arènes | Type | P04783935 | 4999 | - | 10 April, 1913 | P |
|  | Type | P04783936 | - | - | 10 April, 1913 | P |
| *H. luodianensis* S. K. Chen | Holotype | KUN0757302 | 00164 | Luodian, Guizhou | 1 April, 1959 | KUN |
| *H. minor* Dunn | Type | 00071285 | 10792A | - | - | NY |
|  | Type | 00071286 | 10792B | - | - | NY |
|  | Holotype | K000739265 | 10792A | Yunnan | - | K |
|  | Holotype | K000739264 | 10792B | Yunnan | - | K |
|  | Holotype | K000739266 | 10792B | Yunnan | - | K |
|  | Isotype | IBSC0004205 | 62758 | Yunnan | - | IBSC |
| *H. multiflora* F. N. Wei | Type | IBK00190751 | 42678 | Chuozuo, Guangxi | 18 April, 1956 | IBK |
| *H. stenoptera* K.Tan & M.X.Ren | Holotype | - | - | Lushui, Yunnan | - | HUTB |
|  | Isotype | IBK00450922 | 23tk041401 | Lushui, Yunnan | - | IBK |
| *H. tianyangensis* F. N. Wei | Type | IBK00190752 | 53959 | Tianyang, Guangxi | 26 March, 1964 | IBK |
|  | Isotype | KUN0771268 | 53995 | Tianyang, Guangxi | 16 April, 1964 | KUN |
|  | Paratype | IBK00190753 | 123846 | Tianyang, Guangxi | 15 April, 1964 | IBK |
| *H. yunnanensis* Huang ex S. K. Chen | Holotype | KUN0757309 | 0168 | Lushui, Yunnan | 7 April, 1957 | KUN |

**Table S2.** Taxa, GenBank accession numbers, and voucher numbers of *Hiptage* used in this study.

| Species | Locality | GenBank Accession | Voucher Number |
| --- | --- | --- | --- |
| *Hiptage benghalensis* (L.) Kurz | Yangjie, Yunnan, China | MH718400 | M. X. Ren & L. Tang 128 (HUTB) |
|  | Menglian County, Yunnan, China | MH718422 | S. P. Dong 131 (HUTB) |
|  | Daxin County, Guangxi, China | MH718414 | K. Tan & S. P. Dong 95 (HUTB) |
|  | Lekang County, Guizhou, China | MH718415 | K. Tan, S. P. Dong, & M. X. Ren 82 (HUTB) |
|  | Singapore | MH718399 | T. W. Yam 3334 (HUTB) |
|  | Phatthaya, Thailand | MH718408 | K. Tan, S. P. Dong, & M. X. Ren 3344 (HUTB) |
|  | Chiangmai, Thailand | MH718410 | K. Tan, S. P. Dong, & M. X. Ren 3336 (HUTB) |
| *H. bullata* Craib | Lampang, Thailand | MH718412 | K. Tan, S. P. Dong, & M. X. Ren 3320 (HUTB) |
| *H. candicans* Hook. | Chiangmai, Thailand | MH718409 | K. Tan, S. P. Dong, & M. X. Ren 3328 (HUTB) |
|  | Chomthong, Thailand | MH718411 | K. Tan, S. P. Dong, & M. X. Ren 3330 (HUTB) |
| *H. detergens* Craib | KuiBuri, Thailand | MH718404 | K. Tan, S. P. Dong, & M. X. Ren 3328 (HUTB) |
|  | Sam Roi Yot, Thailand | MH718405 | K. Tan, S. P. Dong, & M. X. Ren 3326 (HUTB) |
| *H. ferruginea* Y.H.Tan & Bin Yang | Xishuangbanna, Yunnan, China | MH718402 | S. P. Dong 116 (HUTB) |
|  | Xishuangbanna, Yunnan, China | MH718403 | S. P. Dong 117 (HUTB) |
| *H. incurvatum* K.Tan & M.X.Ren | Pingpo Town, Yunnan, China | MK967956 | K. Tan, H. L. Zheng, & M. X. Ren 201903309 (HUTB) |
| *H. yangshuoensis* K.Tan & K.S.Nguyen | Fuli Town, Yangshuo County, Guilin City, Guangxi, China | PP707781  PP707782 | tk23042201 (IBK) |
| *H. lucida* Pierre | Phatthaya, Thailand | MH718406 | K. Tan, S. P. Dong, & M. X. Ren 38 (HUTB) |
|  | Xishuangbanna, Yunnan, China | MH718418 | Z. N. Qian & S. P. Dong120 (HUTB) |
| *H. luzonica* Merr. | Luzon Island, Philippines | MH718425 | K. Tan, W. Q. Xiang & M. X. Ren 20191181436 (HUTB) |
| *H. marginata* Arènes | Hue, Vietnam | MH718413 | K. Tan & Q. Yang 3363 (HUTB) |
| *H. minor* Dunn | Lekang County, Guizhou, China | MH718398 | K. Tan, S. P. Dong, & M. X. Ren 79 (HUTB) |
|  | Wenshan City, Yunnan, China | MH718423 | K. Tan, S. P. Dong, & M. X. Ren 94 (HUTB) |
|  | Lushui City, Yunnan, China | MH718401 | K. Tan, S. P. Dong, & M. X. Ren 88 (HUTB) |
| *H. monopteryx* Sirirugsa | Phatthaya, Thailand | MH718407 | K. Tan, S. P. Dong, & M. X. Ren 3337 (HUTB) |
| *H. multiflora* F.N.Wei | Nonggang Natural Reserve, Guangxi, China | MH718424  PP707783 | K. Tan & S. P. Dong 52 (HUTB) |
| *H. pauciflora* Y.H.Tan & Bin Yang | Menglian County, Yunnan, China | MH718420 | S. P. Dong 73 (HUTB) |
| *H. stellulifera* Arènes | NhaTrang, Vietnam | MH718429 | K. Tan & S. J. Ling 3376 (HUTB) |
| *H. stenopterum* K.Tan & M.X.Ren | Lushui City, Yunnan, China | OQ968812 | tank 190402001 (HUTB) |
| *H. subglabra* | Nui Chua National Park, Phan Rang, Vietnam | MH718427 | K. Tan & S. J. Ling 3364 (HUTB) |
| *H. tianyangenss* F.N.Wei | Tianyang County, Guangxi, China | MK967960 | K. Tan & S. P. Dong 50 (HUTB) |
| *H. umbellulifera* Arènes | Cana, Phan Rang, Vietnam | MH718426 | K. Tan & S. J. Ling 3386 (HUTB) |
|  | Phan Rang, Vietnam | MH718430 | K. Tan & S. J. Ling 3399 (HUTB) |
|  | Nui Chua National Park, Phan Rang, Vietnam | MH718428 | K. Tan & S. J. Ling 3385 (HUTB) |
| *Heteropterys brunnea* R.Sebast. & Mamede | - | OK284366 | RFAlmeida 579 (HUEFS) |
| *Callaeum psilophyllum* (A.Juss.) D.M.Johnson | - | OK268022 | RFAlmeida 734 (HUEFS) |
| *Niedenzuella multiglandulosa* (A.Juss.) W.R.Anderson | - | OK271417 | RFAlmeida 639 (HUEFS) |
| *Niedenzuella stannea* (Griseb.) W.R.Anderson | - | OK271412 | Pott 1816 (HUEFS) |
